# Microbial Diversity and Quality-Related Physicochemical Properties of Spicy Cabbage in Northeastern China and Their Correlation Analysis

**DOI:** 10.3390/foods11101511

**Published:** 2022-05-23

**Authors:** Lixuan Chang, Guangqing Mu, Mingxu Wang, Tong Zhao, Yanfeng Tuo, Xuemei Zhu, Fang Qian

**Affiliations:** 1 School of Food Science and Technology, Dalian Polytechnic University, No. 1 Qinggongyuan, Ganjingzi District, Dalian 116034, China; changlx0824@163.com (L.C.); gq6552002@aliyun.com (G.M.); a571838513@163.com (M.W.); tyfjq@aliyun.com (Y.T.); zhuxuemei2005@hotmail.com (X.Z.); 2 Dalian Center for Certification and Food and Drug Control, Dalian 116021, China; icp8300@sina.com

**Keywords:** Chinese spicy cabbage, China, microbial diversity, quality, correlation

## Abstract

Chinese spicy cabbage (CSC) is a popular special fermented food in Northeast China. The bacterial community and quality of CSC from different regions of northeastern China (Group_J: Jilin province, Group_L: Liaoning province, Group_H: Heilongjiang province) at retail (Group_P) and home-made (Group_C) were investigated in this study. The determination of the microbial community was achieved using high-throughput sequencing and the quality-related physicochemical characteristics included pH, salinity, total acid (TA), amino acid nitrogen (AAN), reducing sugar (RS), nitrite, and biogenic amines (BAs). Based on OPLS-DA analysis, there was a difference between the quality of Group_C and Group_P. No significant difference was observed in province grouping. Proteobacteria and Firmicutes were the dominant phyla, and the dominant genera were *Lactobacillus*, *Pantoea*, *Weissella*, and *Pseudomonas*. All groups had significant differences in community structure (*p* < 0.05). Compared with Group_C, the relative abundance of opportunistic pathogens (*Pseudomonas* and *Serratia*) in Group_P was lower. *Pseudomonas* and *Serratia* were the biomarkers in Group_H. At the genus level, *Lactobacilluss* and *Weissella* had a positive correlation with pH, Cadaverrine, and salinity (*p* < 0.05), however, they were negatively related to tryptamine. *Pseudomonas* was negatively correlated with salinity (*p* < 0.05). Bacterial community and physicochemical parameters of CSC, as well as the correlation between them, were discussed in this study, providing a reference for future studies on CSC inoculation and fermentation.

## 1. Introduction

Chinese spicy cabbage (CSC) has a unique flavor and is very popular among consumers, with certain health functions, nutritional value, aroma, and taste [1]. CSC is made from Chinese cabbage and various seasonings including fish sauce, chili, garlic, and scallions [2]. The steps in the CSC making are as follows: first, the cabbage is washed and salted for 12 h. Next, excess water is squeezed out of the salted cabbage, and apples, pears, ginger, garlic, and fish sauce are blended and mixed with pepper and fish sauce. The sauce is then spread evenly over the cabbage. Finally, the prepared CSC is cultured at a temperature below 10 °C for 30 days [1].

CSC is frequently home-made or retail-produced, and its quality can be very volatile [1,3] given that the native microorganisms found in the raw materials can vary considerably [4,5]. In fermented foods, nitrite and biogenic amines (BAs) are generated and accumulate, causing food safety issues [6,7]. Nitrite mainly comes from nitrate catalyzes nitrate reductase and is further conversed by nitrite reductase [8,9]. Wang et al. (2018) discovered that microbial communities in paocai are associated with nitrite formation, with nitrite-reducing bacterial genera including *Lactobacillus*, *Pediococcus*, *Acinetobacter*, *Leuconostoc*, *Weissella* [10]. BAs are generally formed from amino acids, which can be formed by amino acid decarboxylases produced by microorganisms [6,9]. Yu et al. (2021) showed that BAs formation was associated with *Leuconostoc*, *Lactobacillus*. and *Pseudomonas* [11]. Total acid (TA), amino acid nitrogen (AAN), and pH are essential indicators that could influence the taste of fermented foods. Lactic acid bacteria (LAB) metabolism is mainly based on reducing sugars (RS) as substrates. The salinity of fermented vegetables is an important indicator of their quality and microbial community [3,8]. Therefore, the quality-related physicochemical indicators, primarily, salinity, pH, AAN, RS, nitrite, and BAs were investigated in this study.

Fermented vegetables are typically made under anaerobic fermentation conditions, and the fermentation process is a complex microbial system involving numerous microorganisms [12]. It is well known that fermented vegetables are mainly controlled by LAB [13]. Many studies have shown that fermented vegetables are contaminated with opportunistic pathogens such as *Pseudomonas*, *Stenostomonas*, and *Serratia* [1,14,15]. As a result, we have investigated the bacterial composition of CSC in this study. The composition of the microbial community structure will also change the dynamics of geographical and climatic conditions, the fermentation process, the type of cabbage and salt used in fermentation, and the microbial community formed by raw materials [16,17,18,19]. Kim et al. (2020) showed that the changes in the microbial diversity were significant in the pre-fermentation period (1, 7, 10d), while at the end of fermentation (30, 40, 50d), there was a significant difference in the relative abundance of Lactobacillus, *Weissella* and *Leuconostoc* [20]. Previous studies have determined that the structure of fermented vegetable flora is similar. At the genus level, the common bacteria are *Lactobacillus*, *Leuconostoc* and *Weiselia*, and some *Pediococcus*, *Stenostomonas* and *Serratia*. At the species level, there are *Lactobacillus sake*, *Lactiplantibacillus pentosus*, and *Lactiplantibacillus plantarum* [4,21]. However, previous studies did not reveal the differences in community structure between retail and home-made CSC, and different provinces in Northeast China. Many kinds of research on fermented vegetables have concentrated on the relationship between bacterial communities and volatile components [15,18,21]. However, flavor substances do not determine the quality of fermented vegetables, and a variety of physicochemical indicators must be investigated to assess the quality of fermented vegetables [22]. As a result, this study examined bacterial communities of various types and provinces, as well as the relationship between bacterial communities and physicochemical parameters of CSC.

To investigate the microbial community in a given sample, culture-independent or culture-dependent methods can be used. MALDI-TOF MS is a culture-dependent method. MALDI-TOF MS enables rapid identification of all colonies present in a culture plate, which has been used to reveal the microbiota in fermented vegetables [23,24]. Based on a culture-independent method, PCR-DGGE was used to study the microbial community of kimchi and sauerkraut [25,26,27,28]. However, due to the limitations of these methods, the microbial flora in fermented foods cannot be gathered entirely. High-throughput sequencing (HTS) is an effective technique for obtaining helpful information about a large microbial diversity. HTS techniques have been widely applied to reveal the microbial diversity of fermented vegetables [29,30].

The aim of this study was to analyze the microbial diversity of 24 samples collected from Northeast China using 16S rRNA and to compare the different groups of CSC. To produce better quality CSC, a correlation analysis of dominant genera and physicochemical parameters (salinity, pH, AAN, RS, nitrite, and BAs) was performed in this study.

## 2. Material and Methods

### 2.1. CSC Samples

24 different CSC samples (30–35 days after fermentation) were collected from three northeastern provinces, 12 of which were sold in the market (purchased from supermarkets or online, branded and labeled) and 12 were homemade samples (purchased from farmers’ markets). Eight came from each of the Jilin, Liaoning, and Heilongjiang provinces (Table 1). Based on the samples collected in this study, they were divided into Group_C (homemade CSC) and Group_P (retail CSC) according to different types, and further divided into Group_L (Jilin province), Group__J (Liaoning province), and Group_H (Heilongjiang provinces) according to provinces. The CSC samples were transported to our laboratory immediately after preparation using an ice cooler stored at −80 °C.

### 2.2. Physicochemical Analysis

#### 2.2.1. pH and TA Measurement

The pH value of the fermentation broth was measured with a pH meter (PHS-25; Shanghai Mettler Company, Shanghai, China).

The content of TA was determined using the method described by Liang et al. (2020) [3]. Accurately weighed 10.00 g samples, soaked in boiling water with 60 mL of distilled water for 30 min, brought the volume to 100 mL, and then filtered. Added 30 mL of deionized water to 10 mL of filtrate, titrated pH to 8.2 ± 0.2 with 0.05 M NaOH, recorded the consumption of sodium hydroxide, blanked 40 mL of deionized water, and repeated the above steps. The TA value was calculated by lactic acid (g/100 mL).

#### 2.2.2. AAN and RS Measurement

AAN content was determined by the method of formol titration described by Zhao et al. (2021) [9]. 36% formalin (10 mL) was added to the above solution at pH 8.2 and titrated to pH 9.2. To the 10 mL of 36% formalin, 40 mL of deionized water was added as a blank control. AAN value was calculated (g/100 g).

The RS content was determined by the DNS method of Yang et al. (2019) [5].

#### 2.2.3. Salinity and Nitrite Measurement

Salt concentration was measured by the salinity option in the conductivity meter (S230-USP/EP conductivity meter; Mettler Company, Shanghai, China) [3].

The nitrite content in CSC was determined by N- (1-naphthalene) ethylenediamine hydrochloride spectrophotometry [31]. Weighed the pounded sample at 5.00 g, added 12.5 mL of saturated borax solution and 60 mL of hot water (70 °C), boiled for 15 min, after cooling, added 1 mL of 106 g/L potassium ferrocyanide solution and 5 mL of 220 g/L zinc acetate solution to mix, kept the volume constant to 100 mL, and then stood for 30 min. The first 20 mL of filtrate was removed, and the remaining 30 mL of supernatant was mixed with 2 mL of 4 g/L p-aminobenzene sulfonic acid solution and 1 mL of 2 g/L N-(1-naphthalene) ethylenediamine dihydrochloride. After 15 min of reaction, the volume was fixed at 50 mL. Absorbance was measured at a 538 nm wavelength. The experiment was repeated three times. Nitrite content is expressed in mg/kg.

#### 2.2.4. BAs Measurement

The determination of BAs was measured according to Li et al. (2022) [6]. 5.00 g samples were vortexed for 30 min with 20 mL of 5% trichloroacetic acid solution, centrifuged at 8000× *g* at 4 °C for 5 min, and the supernatant was collected. Take the precipitate and repeat the above procedure. The supernatant combined volume was determined to be 50 mL with 5% trichloroacetic acid, 10.00 mL filtrate was added with an equal amount of n-hexane, rotated for 5 min, and the organic phase was discarded. 150 μL saturated sodium carbonate solution and 750 μL of dansulfol chloride were added to 750 μL extract. The mixed solution was incubated at 45 °C for 30 min for derivatization. Finally, added 150µL of ammonia water and incubated at 45 °C for 15 min. The resultant sample was filtrated with a 0.22 μm syringe filter.

The detection method was modified according to Li, et al. (2022) and Zhao et al. (2021) [6,9]. The contents of BAs were determined by High Performance Liquid Chromatography (HPLC) (Huapu, S6000, Beijing, China), monitored by a UV detector at 235 nm equipped with a Tnature C18 column (5 μm, 4.6 × 250 mm, Waters Technologies Ireland Ltd., Milford, MA, USA). The flow rate was 1.0 mL/min and the column oven temperature was 40 °C [32]. Acetonitrile and water were used as mobile phases and a gradient mode was programmed shown in Table S1 [33]. The contents of BAs were represented in mg/kg.

### 2.3. DNA Extraction and PCR Amplification for 16S rRNA Sequence

Genomic DNA of the samples was extracted by DNA extraction kit (Magen, Shanghai, China), and the concentration of DNA was detected by agarose gel electrophoresis and NanoDrop 2000. The V3-V4 region was amplified with the 343F-5 primer (5′-TACGGRAGGCAGCAG-3′) and the 798R primer (5′-AGGGTATCTAATCCT-3′), genomic DNA as a template, according to the selection of sequencing region, using the specific primer with Barcode, TKS GFlex DNA Polymerase (Takara, Japan) for PCR, the following PCR conditions were used. The first round of PCR conditions was: initial denaturation at 94 °C for 5 min, 26 cycles of denaturation at 94 °C for 30 s, annealing at 55 °C for 50 s, extension at 72 °C for 30 s, and a final extension at 72 °C for 5 min. The second round of PCR conditions was as follows: initial denaturation at 94 °C for 5 min, 25 cycles of denaturation at 94 °C for 30 s, annealing at 55 °C for 50 s, extension at 72 °C for 30 s, and a final extension at 72 °C for 5 min. Magnetic beads were purified twice, qubit quantification was performed, and high throughput sequencing was performed using an Illumina HiSeq platform (Shanghai OE Biotechnology Co., Ltd., Shanghai, China).

### 2.4. Sequence Data Analysis

Trimmomatic (version 0.35) software was used to scan the raw data sequences. Sequences were cut off at low quality if the sequences with the average quality score were below 20. The flash software (version 1.2.11) was used to splice the qualified double-ended raw data, and the maximum overlap was 200 bp during sequence splicing. The reads with chimera and ambiguous, homologous sequences or below 200 bp were removed using QIIME software (version 1.8.0) [34]. α-diversity was measured by using QIIME software (version 1.8.0). Clean reads were removed from primer sequences and clustered to generate operational taxonomic units (OTU), which were defined by a 97% similarity and selected using Vsearch software (version 2.4.2). Each representative OTU was selected using QIIME package. Silva database (version 132) was annotated and popped using the RDP classifier [35].

### 2.5. Statistical Analysis

Data were analyzed using SPSS 18.0 software (SPSS Inc., Chicago, IL, USA) to perform an analysis of variance (ANOVA) and *t*-test. *p* < 0.05 was considered a significant difference. The boxplot was drawn with Origin 2019b software (OriginLab Inc., Northampton, MA, USA). The linear discriminant analysis (LDA) effect size (LEfSe) and heatmap were plotted by http://www.bioinformatics.com.cn (accessed on 30 March 2022), a free online platform for data analysis and visualization. The OPLS-DA was performed on the SIMCA 13.0 package (Umetrics, Ume, Sweden). Non-metric multidimensional scaling (NMDS) was determined to reflect community structure differences between different groups of samples [30].

## 3. Results

### 3.1. Physicochemical Analysis of CSC

As shown in Table 1, the pH value ranged from 3.93 to 5.53, and the salinity was between 1.73 and 4.32 g/100 g. The AAN and RS ranged from 0.057 to 0.343 g/100 g and 1.16 to 15.35 mg/g, respectively. The TA ranged between 0.09 and 0.6 g/100 g. The nitrite ranged from 0.56 to 4.61 mg/kg. The salinity, nitrite, and RS content were significantly higher in Group_C than in Group_P (*p* < 0.05). There were no significant differences in pH, AAN, and TA between home-made and retail-produced CSC (*p* > 0.05). In province grouping, no significant difference was found in the above physicochemical characteristics (*p* > 0.05). The BAs content of 24 samples is featured in Table 2. In this research, eight different BAs, including tryptamine, phenylethylamine, putrescine, cadaverine, tyramine, histamine, spermine, and spermidine were explored. The content of tryptamine range from 0.02 and 10.54 mg/kg, putrescine was only detected in LJSC2 and LJSC3 at 0.34 mg/kg and 0.74 mg/kg, respectively. It was found that a trace amount of cadaverine was detected in LJC2 and LJSC3. 36.53 mg/kg of histamine was detected only in DLSC2. The contents of tyramine and spermidine were between 1.06–3.07 mg/kg and 0.017–0.038 mg/kg, respectively. The spermine was not discovered in any samples. The contents of total BAs ranged from 0.02 to 50.15 mg/kg. The result showed that the total BAs were significantly higher in Group_C than in Group_P (*p* < 0.05), while there was no significant difference in the province group (*p* > 0.05).

### 3.2. OPLS-DA of Physicochemical of CSC

**Figure 1:**
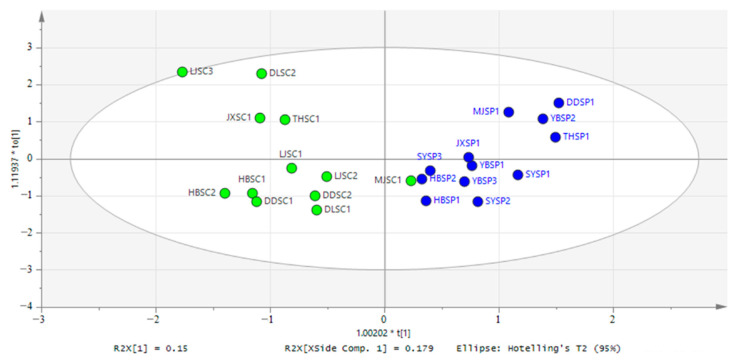
Biplot of the OPLS-DA on the Chemical analysis in different types (A: Group_P: retail packaging, Group_C: home-made, R^2^X = 0.492, R^2^Y = 0.8, Q^2^ = 0.67).

To identify potential differences in physicochemical parameters between groups (different types and different provinces groups), the supervised method OPLS-DA was used. The OPLS-DA score plot (Figure 1) clearly separated Group_ P and Group_ C, with a 49.2% explanatory power for variation in X (R^2^X = 0.492), revealing that the quality of CSC in Group_ P and Group_ C differed. The OPLS-DA results indicated an excellent goodness of fit (R^2^Y = 0.8) and predictability (Q^2^ = 0.67) for the discrimination of different type CSC (Group_ P and Group_C). While in the province grouping, R^2^X and R^2^Y were 0.234 and 0.261 respectively, and Q^2^ was 0.145, indicating the OPLS model was unable to make analytical predictions.

### 3.3. Analysis of α Diversity in CSC

In all samples, the data volume of clean tags was distributed between 60,868 and 73,883, and the data volume of valid tags (the data finally used for analysis) ranged from 59,141 to 71,361 after removing chimera (Table 3). At the 97% sequence similarity level, a total of 5729 OTU numbers were generated in 24 CSC samples (Table 3). As shown in Figure S1, significant differences were observed between samples at various taxonomic levels, implying that diversity varies between samples. The mean numbers of OTUs in Group_P, Group_C, Group_J, Group_L, and Group_H samples were 1102.09, 1192.67, 875.38, 1233.13, and 1183.625 respectively. No significant difference was seen between Group_P and Group_C. The OTUs in Group_J were significantly lower than in Group_H and Group_L (*p* < 0.05), indicating that the bacterial community in Group_ J was the least diverse and species-rich. The Good’s coverage index was above 99.9% in all samples. The sequence depths used were sufficient for the samples in this study. The alpha diversity (Chao 1, Simpson, Observed species, and Shannon) varied across samples, indicating that species diversity and richness varied across 24 samples.

As shown in Figure S2, no significant difference in the Observed species, Chao 1 and Simpson index was observed, among the groups of different types (*t*-test, *p* > 0.05), the Shannon index of Group_P was significantly higher than that of Group_C (*p* < 0.05). The results show that more species can exist as a symbiotic community in Group_P, leading to higher bacterial diversity. Figure S3 indicated that the Chao 1 and Observed species indices of the Group_H and Group_L were significantly higher than those of the Group_J (*p* < 0.05). The Simpson index of Group_H was significantly higher than Group_J in Simpson index (*p* < 0.05). No significant difference was seen in Shannon among the three groups (*p* > 0.05). No significant differences were observed in α diversity between Group_H and Group_L (*p* > 0.05). These revealed that Group_H and Group_L have high community richness.

### 3.4. Bacterial Community of CSC

**Figure 2:**
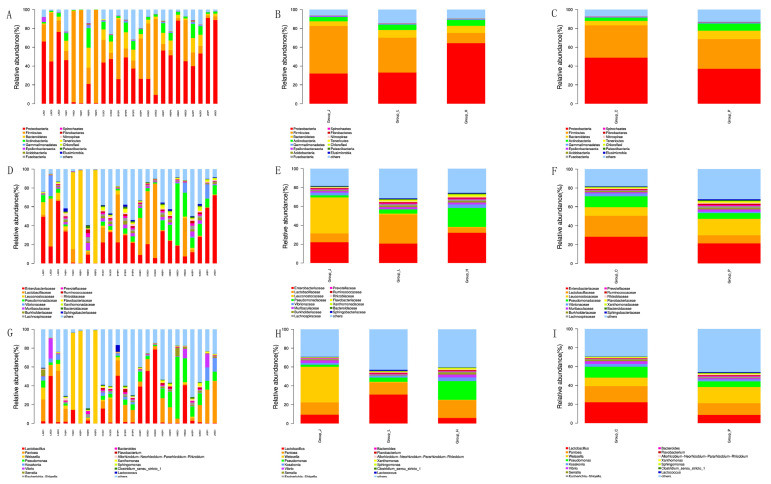
Relative abundance of bacterial communities at phylum (**A**–**C**), family (**D**–**F**), and genus (**G**–**I**) levels in samples of CSC. (Group_P: retail-produced, Group_C: home-made; Group_J: Jilin Province, Group_L: Liaoning Province, and Group_H: Heilongjiang Province).

31 phyla, 316 families, 719 genera, and 1020 species were detected in 24 samples. Figure 2A–C showed the condition of each sample at the phylum level. The three dominant phyla of the CSC microbiota contained the dominant phyla found in this study, including Firmicutes (2.6–98.94%), Proteobacteria (0.059–91.08%), and Bacteroidetes (0.15–21.14%). The average abundance of Firmicutes was 51%, 37%, and 11% in Group_J, Group_L, and Group_H and 34.24% and 31.6% in the Group_P and Group_C, respectively. The relative abundance of Firmicutes was the most abundant phylum in the samples, followed by Actinobacteria, Gemmatimonadetes, Acidobacteria, Fusobacteria, and Epsilonbacteraeota. Meanwhile, at the family level (Figure 2D–F), the dominant families were Lactobacillaceae (0.03–78.54%), Leuconostocaceae (0.03–8.76%), and Enterobacteriaceae (0.2–72.11%). Pseudomonadaceae (0.04–65.48%), Vibrionaceae (0.04–21.85%), Muribaculaceae (0.04–7.66%) and Burkholderiaceae (0.02–3.74%) followed these. The genera *Lactobacillus* (0.04–78.54%), *Panthenia* (0.08–54.61%) and *Weiselia* (0.03–98.71%) were dominant (Figure 2G–I) and also include *pseudomonas* (0.04–65.48%), *Kosakonia* (0.01–14.36%), *Vibrio* (0–21.21%), *Serratia* (0.01–8.80%), *Bacteroides* (0.02–3.09%), *Flavobacterium* (0–3.35%) and *Xanthomonas* (0–2.26%). *Lactobacillus*, *Pseudomonas*, *Serratia*, and *Vibrio* were found in greater abundance in Group_C. *Weisseria* (37.9%) and *Serratia* (0.96%) had higher mean relative abundances in Group_J than in Group_L (0.83% and 0.38%) and Group_H (0.51% and 0.22%). *Lactobacillus* was found in higher concentrations in Group_L (30.7%) than in Group_J (9.34%) and Group_H (5.87%). The mean relative abundance of *Weisseria* (37.9%) and *Serratia* (0.96%) in Group_J was higher than that in Group_L (0.83% and 0.38%) and Group_H (0.51% and 0.22%). *Pseudomonas* and *vibrio* were more prevalent in Group_H (19.9% and 3.05%) than in Group_J (2.1% and 2.8%) and Group_L (4.48% and 0.12%). *Lactobacillus*, *Pantoea*, and *Pseudomonas* were the most important genera in Group_C, while *Lactobacillus*, *Pantoea*, and *Weiselia* were dominant in Group_P. The dominant genera in the provincial grouping were as follows: *Lactobacillus*, *Pantoea*, and *Weiselia* were in Group_J, *Lactobacillus*, *Pantoea*, and *Pseudomonas* were in Group_L, *Pseudomonas Pantoea* and *Lactobacillus* were in Group_H. The results showed that there were differences in 15 major phyla, families, and genera in different groups. The relative abundance of opportunistic pathogens in Group_P was lower than in Group_C, indicating that the retail-produced CSC was safer.

### 3.5. Multivariate Analysis of the CSC Bacterial Community

**Figure 3:**
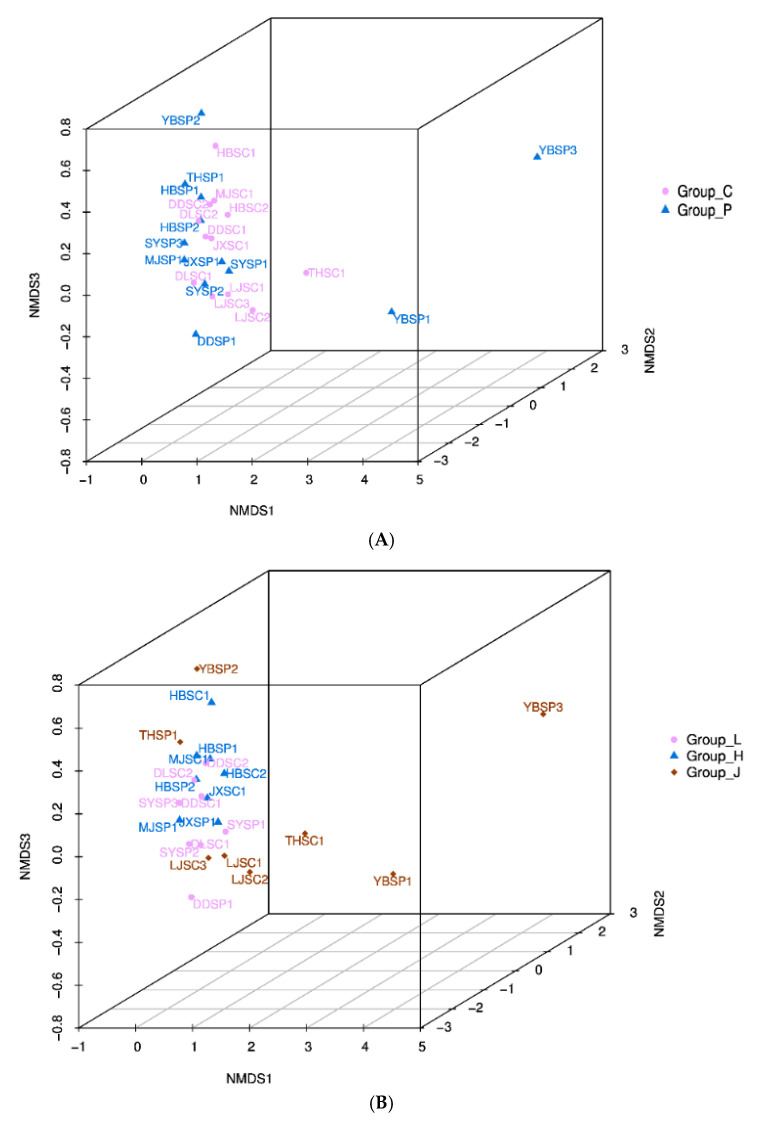

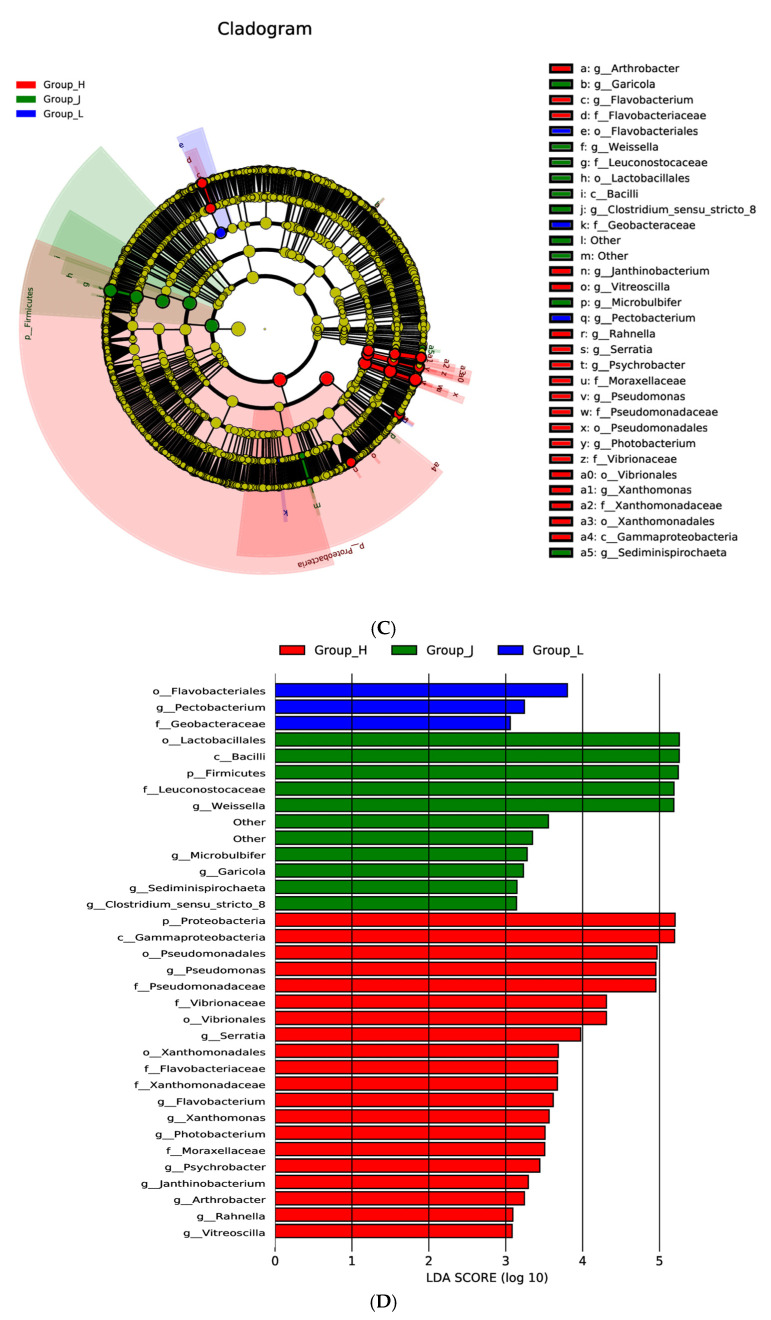
(**A**,**B**): Non-metric multi-dimensional scaling (NMDS) of the data set (24 samples). LEFSE (**C**) and Linear discriminate analysis (**D**) of the bacterial community in the CSC of different provinces (Group_P: retail- produced, Group_C: home-made; Group_J: Jilin Province, Group_L: Liaoning Province, and Group_H: Heilongjiang Province).

To determine whether there was a significant correlation between the microbial communities of different groups, NMDS analysis was performed on the β diversity of the samples (Figure 3). As shown in Figure 3A, there was some overlap between Group_C and Group_P in the NMDS analysis, but the microbiota in Group_C samples was more similar to each other than those in Group_P samples, indicating that the differences in microbiota were less significant in Group_C than in Group_P. Figure 3B showed that significant differences were found in microbial community structure between Group_J and both Group_H and Group_L (*p* < 0.05), but that the bacterial community structure of Group_H and Group_L was similar.

LEfSe was used to identify the differences in relative microbial abundance between Group_J, Group_L, and Group_H. As shown in Figure 3C, a total of 30 branches including 2 orders, 2 phyla, 4 families, 16 genera, and 12 species were found. As shown in Figure 3D, there were 3 taxa in Group_L, 11 taxa in Group_ J, and 20 taxa in Group_H. Geobacteraceae, Leuconostocaceae, and Pseudomonadaceae were the biomarkers at the family level in Group_L, Group_J, and Group_H, respectively. At the genus level, Group_ L had more *Pectobacterium*, *Weissella* was the most abundant in Group_J, and Group_ H had a higher relative population of *Pseudomonas* and *Serratia*. The results showed that the pathogenic microorganisms were mainly distributed in Group_H.

### 3.6. Association between Bacteria and Physicochemical Factors in CSC

**Figure 4:**
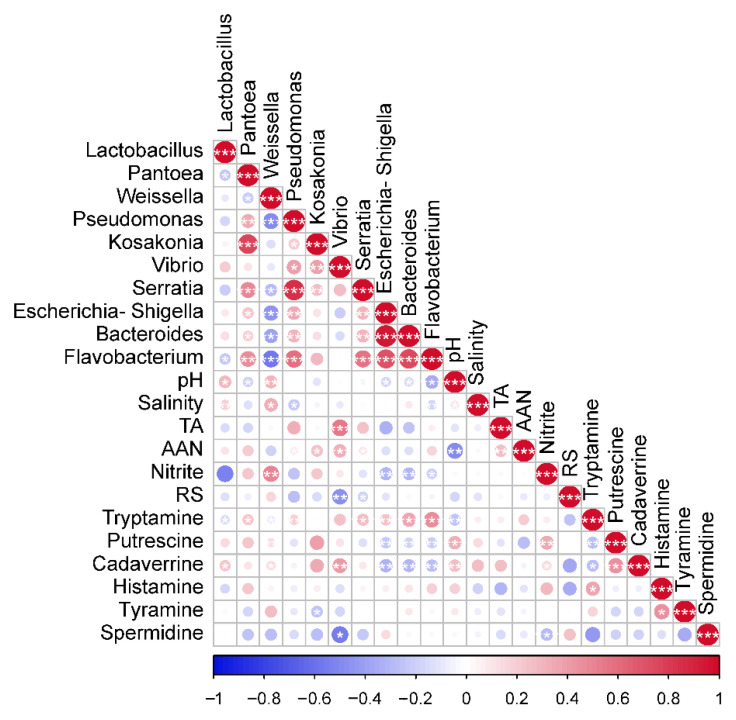
Spearman rank correlation analysis to study the physicochemical factors and dominant genus in Chinese fermented vegetable. The Spearman correlation coefficient r ranges from −0.5 to 0.5, r < 0 is a negative correlation, r > 0 is a positive correlation. * *p* < 0.05, ** *p* < 0.01, *** *p* < 0.001.

Figure 4 depicted the correlations between physicochemical factors and dominant genus in CSC samples. At the genus level, *Lactobacillus* and *Weissella* had a positive correlation with pH, Cadaverrine, and salinity (*p* < 0.05), however, they negatively related with Tryptamine. *Weissella* was positively correlated with nitrite and Putrescine. *Vibrio* was positively correlated with TA, AAN, and Cadaverrine, while negatively related with RS and Spermidine. *Escherichia-Shigella*, *Flavobacterium*, and *Bacteroides* indicated opposite correlations to the relationship with nitrite, Putrescine, and Cadaverrine. Flavobacterium and *Pseudomonas* were negatively realted with salinity (*p* < 0.05). Overall, the results show that salinity, AAN, pH, RS nitrite, BAs, and TA were important parameters affecting the microbial community.

## 4. Discussion

This study compared the microbial diversity and quality of different types CSC from different provinces in Northeast China and studied the correlation between the relative abundance of genus level and physicochemical parameters.

Not only do pH and TA determine the key variables of the fermentation degree but they also affect the taste of the pickle. The results showed that the content of nitrite in all samples was less than 20 mg/kg, which was stipulated by the Chinese national standard as the highest level of contaminants allowed in foods [8]. Nitrite was a recognized carcinogen and was considered an important indicator of food safety. It is mainly produced by nitrate-reducing bacteria in the early stage of fermentation [36]. Yang et al. (2020a) found that as fermentation progresses, the acid produced by LAB will inhibit the nitrate-reducing bacteria, thus reducing the content of nitrite [7]. Therefore, the nitrite content in all samples is less than 5 mg/kg.

As an indicator of CSC nutrition and sensory quality, AAN has an important impact on the flavor of a product. During the fermentation process, the protein was hydrolyzed into various amino acids, which give paocai its good flavor [37]. JXSC1 has a higher number of AAN. Jeotgal is rich in amino acids and plays an important role in the unique flavor of pickles [30]. High Jeotgal content will lead to an increase in AAN content. Therefore, the result was due to the fact that JXSC1 had a high content of fish sauce.

BAs are nitrogen-containing compounds that are mainly produced by microorganisms during fermentation through the transaminase action of amino acid decarboxylase. Many studies have shown that a high concentration of BAs may lead to poisoning [38]. Therefore, BAs in CSC were discussed in this study. BAs were presented in large quantities in DLSC2, and the content of histamine reached 35.63 mg/kg, which was much lower than the toxicity limit of (100 mg/kg) recommended by Santos et al. (1996) [39]. According to Jeong & Lee (2015) and Kim & Kim (2014b), a trace amount of BAs produced from kimchi were from the *Weiselia*, *Leuconostoc*, and *Lactobacillus* [40,41]. One reason for that phenomenon was that fish sauce in kimchi produced BAs such as histamine and tyramine [42,43,44]. The total BAs content range, from 0–50.15 mg/kg in our experiment, was lower than the previous literature, which has suggested limits for BAs content in food products of 1000 mg/kg for total BAs content [45]. As a result, the samples collected are relatively safe; humans and animals can easily break down a small amount of BAs in their bodies. The total BAs were significantly higher in Group_C than Group_P (*p* < 0.05), while there was no significant difference in the province group (*p* > 0.05). The above results indicated that there were potential food safety risks in the CSC of Group_C.

The α-diversity was represented by Chao1, Shannon, Observedspecies, and the Simpson index, which were used to assess community richness and evenness [14]. Interestingly, lower bacterial diversity was discovered in home-made CSC, which could be attributed to the home-made CSC’s unfavorable environment, where nutrients are depleted and competition between microbes results in fewer microorganisms coexisting [1,46].

Previous research and this study reported that Firmicutes and Proteobacteria were the major phyla in fermented vegetables. The dominant genus of CSC may differ in different groups. *Lactobacillus*, *Pantoea*, and *Pseudomonas* were the key genus in CSC samples. *Lactobacillus* was the most dominant bacterium in the whole fermented vegetable industry in China, such as Northeast suan-cai, Sichuan pickle, and southwest pickle [18,47]. Jung (2018) and Yu (2021) found that *Pantoea* was in fermented mustard and jeotgal, which indicated that the bacteria community was related to the raw material (CSC contains jeotgal) [11,48]. As shown in Figure 2G, because *Pseudomonas* is an aerobic bacterium, homemade CSC conditions can disrupt the fermentation process’s anaerobic environment, leading to an increase in the number of *Pseudomonas* [14]. Contamination by genera *Pseudomonas* has been identified in previous studies on paocai and fermented cucumbers [19,49,50]. The results of this study were consistent with previous studies. *Leuconostoc* was also a dominant bacteria in CSC, which was not detected in this research.

Surprisingly, a relatively high abundance of *vibrio* was found in a few samples (LJSC1 and HBSC1). Jung et al. (2013) and Hong et al. (2014) found that *vibrio* was in both Jeotgal and salted Chinese cabbage [51,52]. The *vibrio* primarily came from the raw shrimp flora of Jeotgal, a CSC ingredient [36]. It is a gram-negative bacteria that can cause epidemics and is associated with gastroenteritis [53]. The relative abundance of *Serratia* was higher in LJSC1 and HBSC1. *Serratia* belongs to the family of Enterobacteriaceae, which can cause respiratory tract infections, intravenous catheter-associated infections, urinary tract infections, pneumonia, septicemia, meningitis, endocarditis, and wound infections [54,55,56,57]. Previous literature has shown that *Serratia* is also present in fermented vegetables [34,47,58]. The phenomenon indicated that the environment and conditions for home-made CSC were harmful. These results suggested the potential microbial safety and spoilage risks of home-made CSC. Therefore, it is important to reduce the content of opportunistic bacteria and harmful microorganisms in CSC. There were no official limitations on *Serratia*, *Pseudomonas*, and *Vibrio* in CSC in China at the time. However, according to local Chinese standards (DB22/T 1758-2012), *Shigella*, *Salmonella*, and *Staphylococcus aureus* should not be found in CSC. There were some differences in the bacterial community structure among different groups of samples, which were due to the raw materials, environment, climate, and region [59,60]. Complex microbial community profiles were identified using LEfSe, an algorithm for finding high-dimensional biomarkers and quantifying the genetic traits that characterize biological samples, to obtain insight into the ecosystem of CSC [22]. The LEfSe data revealed significant changes in microbial composition for geographic groups (Group_L, Group_J, and Group_H), implying that the bacterial community is influenced by the environment and temperature [29]. To summarize, Firmicutes, Enterobacteriaceae and *Leuconostocaceae*, and *Lactobacillus* and *Pantoea* were the dominant bacteria at the phyla, family, and genus levels, respectively (Figure 2), with significant variances in relative abundance between these bacteria, indicating that sample species diversity was varied (consistent with the results of the alpha diversity analysis) [22]. It also implied that the microbial composition of each CSC sample was relatively similar, which was consistent with the β-diversity analysis results (Figure 3).

Salinity is one of the important factors affecting the microbial community. As reported by Liu et al. (2019), the pathogens were sensitive to salt, for example, the salinity showed a negative correlation with the content of *Pseudomonas caeni*. We came to a consistent conclusion [1]. However, high salt content may inhibit the growth of certain LAB [34], contrary to our results (Figure 4), but we deduced that this was due to the low salinity of the samples collected (the mean value of salinity was 2.5%), *Lactobacillus* and *Weissella* were able to grow under low salt conditions. *Lactobacillus* was able to produce trace amounts of BAs and also to degrade them, which is consistent with the results of previous studies [6,40,41]. The positive correlation between *Vibrio* and Cadaverrine could be attributed to high levels of fish sauce, which would result in increased BAs, as well as the raw shrimp taxa in the fish sauce, which would lead to an increase in the relative abundance of *Vibrio* [36,42]. Bacteria from the *Escherichia-Shigella* group were also found in the denitrifying population, implying that the *Escherichia-Shigella* can prevent nitrate reduction to nitrite, so the *Escherichia-Shigella* was negatively correlated with nitrite [40,61]. Early literature has shown that LAB (such as *Lactobacillus*, *Pediococcus*, *Weissella*, and others) can degrade nitrite, but we discovered the opposite [7,10,13]. This implies that our subsequent experiments will require a nitrite-degrading screen LAB. Increased TA in CSC has been linked to the production of organic acids (acetic acid and lactic acid) by LAB [1,3]. Although Cao et al. (2017) discovered that high acidity inhibited Serratia growth, we found the opposite [14]. We speculated that this was due to the low acidity of the samples, which did not inhibit the growth of *Pseudomonas*, *Vibrio*, and *Serratia*. It is crucial to comprehend the makeup and variety of CSC bacteria communities, as well as the links between microbial communities and physicochemical features.

Overall, future research should focus on the isolation of LAB from CSCs with opportunistic infection suppression and nitrite degradation to improve the quality of fermented vegetable products.

## 5. Conclusions

In this study, the microbial diversity and physicochemical characteristics of CSC in Northeast China were explored, and the correlation between major genera and quality-related physiochemical properties was researched. Proteobacteria and *Lactobacillus* were the most common bacteria at both the phyla and genus levels. The relative abundance of opportunistic pathogens such as *Serratia*, *Vibrio*, and *Pseudomonas* was higher in Group_C than in Group_P. At the genus level, the biomarkers of Group_L and Group_J were *Pectobacterium* and *Weissella*, respectively, and Group_H was *Pseudomonas* and *Serratia*. *Serratia*, *Vibrio*, *Pseudomonas*, *Escherichia-Shigella*, *Kosakonia*, and *Flavobacterium* were found to have significant correlations with the majority of measured physiochemical characteristics, including salinity, AAN, pH, RS, BAs, nitrite, and TA. Because of the strong correlations, several potential pathogenic bacteria in home-made CSC should be controlled. Overall, understanding the microbes of CSC, as well as the correlations between microbial and physicochemical properties, is critical for future industrial production of high-quality CSC.

## Figures and Tables

**Table 1:** Physicochemical properties of the CSC samples.

Sample	Type	Sampling Province	Sampling Location	pH	Salinity (g/100 g)	AAN (g/100 g)	TA (g/100 g)	Nitrite (mg/kg)	RS (mg/g)
LJSC1	homemade	Jilin	Linjiang	5.36 ± 0.03 ^c^	4.32 ± 0.06 ^a^	0.136 ± 0.007 ^e,f,g^	0.21 ± 0.01 ^i^	2.00 ± 0.18 ^f^	3.24 ± 0.16 ^k,l^
LJSC2	homemade	Jilin	Linjiang	4.75 ± 0.03 ^g^	2.43 ± 0.24 ^h^	0.150 ± 0.01 ^d,e^	0.23 ± 0.02 ^g,h^	2.79 ± 0.12 ^e^	1.16 ± 0.02 ^o^
LJSC3	homemade	Jilin	Linjiang	5.87 ± 0.02 ^a^	2.98 ± 0.01 ^e,f^	0.053 ± 0.001 ^l^	0.09 ± 0.01 ^k^	4.61 ± 0.09 ^a^	7.72 ± 0.24 ^b^
THSC1	homemade	Jilin	Linjiang	5.08 ± 0.03 ^e^	1.96 ± 0.02 ^m^	0.057 ± 0.006 ^j,k^	0.19 ± 0.01 ^i^	0.57 ± 0.04 ^k^	0.47 ± 0.011 ^p^
THSP1	retail	Jilin	Tonghua	4.81 ± 0.03 ^f^	1.99 ± 0.02 ^l,m^	0.095 ± 0.000 ^i^	0.32 ± 0.01 ^f^	4.04 ± 0.14 ^k,l^	3.35 ± 0.08 ^k,l^
YBSP1	retail	Jilin	Yanji	4.13 ± 0.03 ^m^	1.93 ± 0.06 ^m,n^	0.127 ± 0.000 ^g^	0.14 ± 0.001 ^j^	0.72 ± 0.11 ^i,j,k^	6.54 ± 0.04 ^d^
YBSP2	retail	Jilin	Yanji	4.65 ± 0.06 ^i^	1.73 ± 0.03 ^o,p^	0.061 ± 0.001 ^k,l^	0.12 ± 0 ^j,k^	0.63 ± 0.05 ^j,k,l^	4.05 ± 0.02 ^g,h^
YBSP3	retail	Jilin	Yanji	4.33 ± 0.03 ^k^	2.08 ± 0.01 ^j,k,l,m^	0.160 ± 0.004 ^i,j^	0.25 ± 0.01 ^g^	0.95 ± 0.04 ^h,i^	3.70 ± 0.15 ^i,j^
DLSC1	homemade	Liaoning	Dandong	3.93 ±0.02 ^n^	3.31 ± 0.05 ^c^	0.074 ± 0.001 ^j,k^	0.47 ± 0.09 ^c^	1.96 ± 0.15 ^f^	5.05 ± 0.07 ^f^
DLSC2	homemade	Liaoning	Dandong	4.74 ± 0.06 ^g,h^	1.65 ± 0.02 ^p^	0.092 ± 0.005 ^i^	0.34 ± 0.03 ^e,f^	2.88 ± 0.21 ^e^	6.77 ± 0.08 ^c^
DDSC1	homemade	Liaoning	Dalian	4.27 ± 0.04 ^l^	1.81 ± 0.02 ^n,o^	0.137 ± 0.005 ^i^	0.52 ± 0.037 ^b^	4.30 ± 0.16 ^b^	3.18 ± 0.21 ^l^
DDSC2	homemade	Liaoning	Dalian	4.15 ± 0.03 ^m^	1.70 ± 0.03 ^o,p^	0.131 ± 0.003 ^i,j^	0.44 ± 0.01 ^c^	3.44 ± 0.01 ^d^	4.01 ± 0.08 ^g,h^
SYSP1	retail	Liaoning	Shenyang	4.24 ± 0.02 ^l^	2.14 ± 0.01 ^j,k,l^	0.098 ± 0.011 ^h,i^	0.39 ± 0.02 ^d^	0.62 ± 0.08 ^j,k,l^	4.84 ± 0.06 ^f^
SYSP2	retail	Liaoning	Shenyang	4.39 ±0.02 ^j,k^	3.18 ± 0.01 ^c,d^	0.085 ± 0.003 ^e,f,g^	0.60 ± 0.03 ^a^	0.56 ± 0.09 ^k,l^	3.30 ± 0.16 ^k,l^
SYSP3	retail	Liaoning	Shenyang	4.69 ± 0.02 ^h,i^	2.92 ± 0.01 ^f^	0.112 ± 0.008 ^g^	0.37 ± 0.03 ^d,e^	1.01 ± 0.02 ^h^	4.11 ± 0.04 ^g^
DDSP1	retail	Liaoning	Dandong	5.19 ± 0.08 ^d^	2.29 ± 0.17 ^i^	0.057 ± 0006 ^l^	0.19 ± 0.01 ^i^	0.42 ± 0.04 ^l^	3.86 ± 0.27 ^h,i^
HBSC1	homemade	Heilongjiang	Haerbin	4.37 ± 0.03 ^j,k^	3.56 ± 0.15 ^c^	0.091 ± 0.010 ^h^	0.12 ± 0.05 ^j,k^	2.19 ± 0.06 ^f^	3.97 ± 0.12 ^g,h^
HBSC2	homemade	Heilongjiang	Haerbin	4.41 ± 0.02 ^j^	3.27 ± 0.21 ^c^	0.083 ± 0.014 ^f^	0.14 ± 0.001 ^j^	3.82 ± 0.18 ^c^	2.11 ± 0.10 ^m^
JXSC1	homemade	Heilongjiang	Jixi	5.04 ± 0.03 ^e^	2.03 ± 0.01 ^k,l,m^	0.230 ± 0.019 ^b^	0.15 ± 0.01 ^j^	2.67 ± 0.28 ^e^	15.35 ± 0.19 ^a^
MJSC1	homemade	Heilongjiang	Mudanjiang	5.53 ± 0.04 ^b^	2.17 ± 0.02 ^i,j,k^	0.343 ± 0.004 ^a^	0.15 ± 0.01 ^j^	0.64 ± 0.03 ^j,k,l^	3.46 ± 0.19 ^j,k^
JXSP1	retail	Heilongjiang	Mudanjiang	4.70 ± 0.02 ^g,h,i^	3.11 ± 0.01 ^d,e^	0.175 ± 0.003 ^c^	0.25 ± 0.02 ^g^	1.48 ± 0.04 ^g^	5.28 ± 0.10 ^e^
MJSP1	retail	Heilongjiang	Jixi	5.09 ± 0.03 ^e^	2.21 ± 0.07 ^i,j^	0.061 ± 0.003 ^k,l^	0.14 ± 0.02 ^j^	1.26 ± 0.03 ^g^	1.85 ± 0.01 ^n^
HBSP1	retail	Heilongjiang	Haerbin	4.35 ± 0.04 ^j,k^	2.69 ± 0.05 ^g^	0.145 ± 0.023 ^d,e,f^	0.42 ± 0.06 ^c^	1.44 ± 0.02 ^g^	3.45 ± 0.04 ^j,k^
HBSP2	retail	Heilongjiang	Haerbin	4.66 ± 0.03 ^i^	2.75 ± 0.01 ^g^	0.133 ± 0.023 ^f,g^	0.35 ± 0.03 ^ef^	0.88 ± 0.04 ^h,i,g^	3.48 ± 0.06 ^j,k^

^a–p^ Means ± SD for three experiments with different letters indicate the significant difference in physicochemical properties (*p* < 0.05, ANOVA, Duncan-LSD).

**Table 2:** Contents of BAs in CSC (mg/kg).

Sample	Tryptamine	Phenethylamine	Putrescine	Cadaverrine	Histamine	Tyramine	Spermidine	Spermine	Total Amine
LJSC1	ND	ND	ND	0.76 ± 0.06 ^a,b^	ND	ND	ND	ND	0.76 ± 0.06 ^h,i^
LJSC2	ND	ND	0.34 ± 0.03 ^b^	1.02 ± 0.37 ^a^	ND	ND	ND	ND	1.36 ± 0.40 ^f,g^
LJSC3	ND	ND	0.74 ± 0.004 ^a^	ND	ND	ND	ND	ND	0.74 ± 0.00 ^h,i^
THSC1	4.83 ± 0.11 ^c^	ND	ND	ND	ND	2.31 ± 0.2 ^b^	ND	ND	7.13 ± 0.14 ^c^
THSP1	ND	ND	ND	ND	ND	ND	0.017 ± 0.00 ^c^	ND	0.02 ± 0.00 ^j^
YBSP1	ND	ND	ND	ND	ND	ND	0.019 ± 0.00 ^c^	ND	0.02 ± 0.00 ^j^
YBSP2	0.95 ± 0.49 ^e,f^	ND	ND	ND	ND	ND	ND	ND	0.95 ± 0.49 ^h^
YBSP3	ND	ND	ND	ND	ND	1.41 ± 0.01 ^d^	ND	ND	1.41 ± 0.01 ^g,h^
DLSC1	9.38 ± 0.247 ^b^	ND	ND	ND	ND	ND	ND	ND	9.38 ± 0.24 ^b^
DLSC2	10.55 ± 1.44 ^a^	ND	ND	ND	36.53 ± 0.173 ^a^	3.07 ± 1.45 ^a^	ND	ND	50.15 ± 0.2 ^a^
DDSC1	0.04 ± 0.024 ^f,g^	ND	ND	ND	ND	ND	ND	ND	0.04 ± 0.02 ^i^
DDSC2	ND	ND	ND	ND	ND	ND	0.018 ± 0.00 ^c^	ND	0.02 ± 0.00 ^j^
SYSP1	ND	ND	ND	ND	ND	ND	0.038 ± 0.01 ^a^	ND	0.04 ± 0.09
SYSP2	ND	ND	ND	ND	ND	ND	0.016 ± 0.00 ^c^	ND	0.16 ± 0.00 ^j^
SYSP3	ND	ND	ND	ND	ND	1.76 ± 0.03 ^b,c^	ND	ND	1.76 ± 0.03 ^e^
DDSP1	ND	ND	ND	ND	ND	ND	0.032 ± 0.01 ^b^	ND	0.03 ± 0.01 ^j^
HBSC1	0.82 ± 0.11 ^e,f^	ND	ND	ND	ND	ND	ND	ND	0.817 ± 0.01 ^h^
HBSC2	0.02 ± 0.002 ^e^	ND	ND	ND	ND	ND	0.020 ± 0.03 ^c^	ND	0.04 ± 0.10 ^j^
JXSC1	0.42 ± 0.04 ^f,g^	ND	ND	ND	ND	ND	ND	ND	0.42 ± 0.04 ^i^
MJSC1	0.37 ± 0.06 ^f,g^	ND	ND	ND	ND	1.06 ± 0.01 ^d^	ND	ND	1.44 ± 0.01 ^e,f^
JXSP1	ND	ND	ND	ND	ND	1.41 ± 0.11 ^c,d^	ND	ND	1.41 ± 0.11 ^f^
MJSP1	2.27 ± 0.27 ^d^	ND	ND	ND	ND	ND	ND	ND	2.27 ± 0.27 ^d^
HBSP1	1.33 ± 0.09 ^e^	ND	ND	ND	ND	ND	ND	ND	1.33 ± 0.10 ^f,g^
HBSP2	ND	ND	ND	ND	ND	ND	ND	ND	ND

^a–j^ Means ± SD (standard deviation) for three experiments with different letters indicate the significant difference in BAs content (*p* < 0.05, ANOVA, Duncan-LSD). ND: not detected.

**Table 3:** Number of clean tags, Good’s coverage, observed OTUs, and alpha diversity estimators of 24 CSC samples.

Samples	Clean Tags	Valid Tags	OTU	Goods Coverage	Chao1	Shannon	Observed Species	Simpson
LJSC1	73,883	66,102	948	1.00	1146.65	4.96	920.20	0.92
LJSC2	73,442	67,242	790	1.00	1070.54	2.94	750.50	0.70
LJSC3	68,440	63,119	1228	1.00	1349.10	4.30	1213.00	0.73
THSC1	71,936	67,339	624	1.00	943.30	1.21	584.70	0.32
THSP1	71,804	66,850	1383	1.00	1536.54	5.77	1360.9	0.9
YBSP1	60,868	59,141	400	1.00	764.40	0.28	399.70	0.04
YBSP2	71,639	65,163	1284	1.00	1401.83	8.46	1271.5	0.98
YBSP3	73,123	71,361	346	1.00	682.16	0.20	308.50	0.03
DLSC1	71,531	65,072	1413	1.00	1527.51	7.38	1398.70	0.97
DLSC2	72,021	65,941	1270	1.00	1452.50	5.76	1251.50	0.89
DDSC1	72,149	65,970	1291	0.99	1492.86	3.52	1259.00	0.67
DDSC2	71,607	67,690	1100	1.00	1239.84	2.23	1070.60	0.38
SYSP1	71,279	66,859	931	1.00	1098.90	3.34	901.40	0.72
SYSP2	72,406	66,156	1294	1.00	1419.27	7.5	1278.30	0.96
SYSP3	70,810	65,458	1406	1.00	1571.20	6.23	1387.20	0.88
DDSP1	71,945	67,382	1160	1.00	1266.26	4.66	1141.60	0.81
HBSC1	71,905	65,058	1041	1.00	1197.20	3.58	1021.10	0.66
HBSC2	71,596	64,107	934	1.00	1122.21	3.40	915.60	0.77
JXSC1	73,590	65,350	1157	0.99	1371.42	4.71	1127.30	0.83
MJSC1	71,864	65,526	1309	1.00	1434.70	6.64	1294.80	0.94
JXSP1	73,422	63,327	961	0.99	1233.36	4.65	938.30	0.87
MJSP1	70,959	64,331	1298	1.00	1492.25	7.89	1285.30	0.98
HBSP1	72,175	66,277	1420	1.00	1632.38	6.74	1396.60	0.94
HBSP2	72,303	65,943	1349	1.00	1522.56	6.14	1330.50	0.92

## Data Availability

The data presented in this study are available in this article and supplementary materials.

## Supplementary Materials

The following supporting information can be downloaded at: https://www.mdpi.com/article/10.3390/foods11101511/s1, Figure S1: 24 CSC samples annotated under classification level on OTU levelbar plot; Figure S2: α diversity index of spicy Chinese cabbage samples with different types (Group_P: retail packaging, Group_C: home-made). (A) Chao 1, (B) observed_species, (C) simpson, (D) Shannon; Figure S3: α diversity index of spicy Chinese cabbage samples with different province (Group_J: Jilin Province, Group_L: Liaoning Province, and Group_H: Heilongjiang Province). (A) Chao 1, (B) observed_species, (C) simpson, (D) Shannon; Table S1: The gradient elution program.
