# Supplementary material for: Microbial Diversity and Quality-Related Physicochemical Properties of Spicy Cabbage in Northeastern China and Their Correlation Analysis
Source: Foods. 2022 May 23;11(10):1511. doi: 10.3390/foods11101511 (PMC9141884; doi:10.3390/foods11101511)
Supplement: Supplementary file 1 [file foods-11-01511-s001.zip › foods-1688262-supplementary.pdf]

foods

MDPI

Supplementary material

Microbial Diversity and Quality-Related Physicochemical Properties of Spicy Cabbage in Northeastern China and Their Correlation Analysis

Lixuan Chang [1], Guangqing Mu [1], Mingxu Wang [1], Tong Zhao [2], Yanfeng Tuo [1], Xuemei Zhu [1] and Fang Qian [1,3,*]

1 School of Food Science and Technology, Dalian Polytechnic University, Dalian 116034, China; changlx0824@163.com (L.C.); gq6552002@aliyun.com (G.M.); a571838513@163.com (M.W.); tyfjq@aliyun.com (Y.T.); zhuxuemei2005@hotmail.com (X.Z.)
2 Dalian Center for Certification and Food and Drug Control, Dalian 116021, China; icp8300@sina.com
3 School of Food Science and Technology, Dalian Polytechnic University, No. 1 Qinggongyuan, Ganjingzi District, Dalian 116034, China
* Correspondence: qf09@163.com; Tel.: +86-13130035327; Fax: +86 41186322121

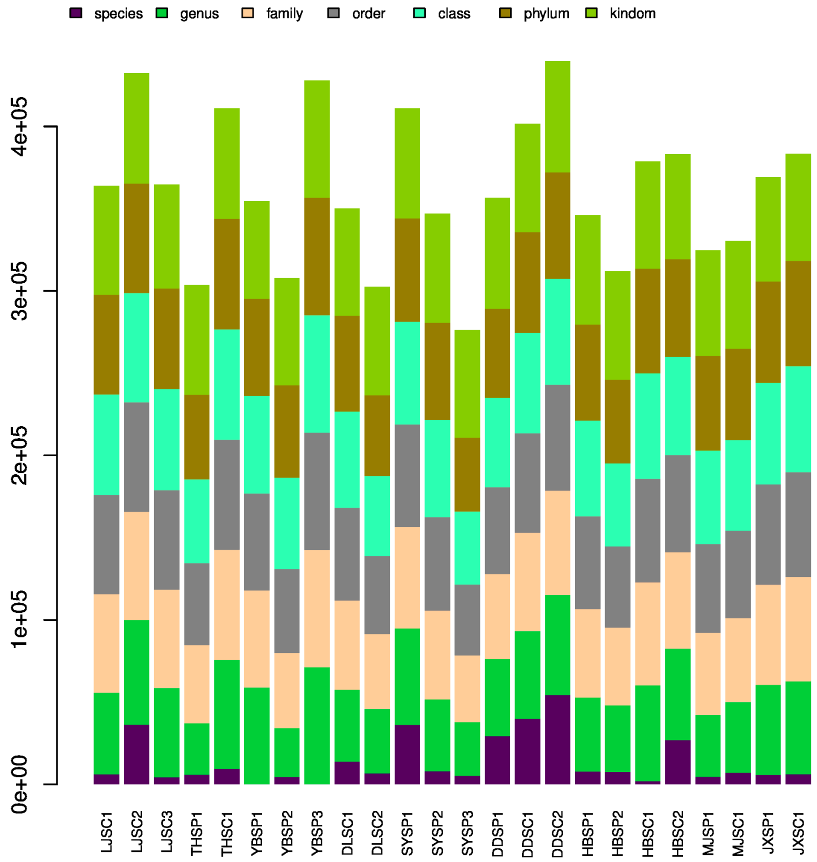

Figure S1. 24 CSC samples annotated under classification level on OTU levelbar plot.

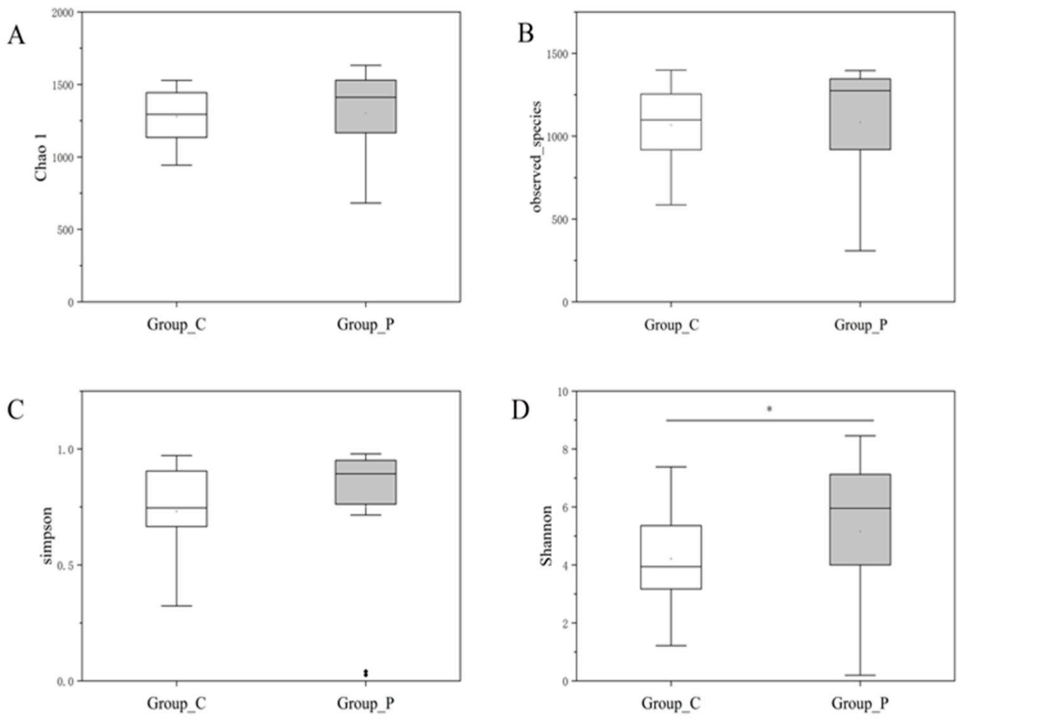

Figure S2. α diversity index of spicy Chinese cabbage samples with different types (Group_P: retail packaging, Group_C: home-made). (**A**) Chao 1; (**B**) observed_species; (**C**) simpson; (**D**) Shannon. * denote significant differences ($p < 0.05$, T-test, not significant). The box plots show the smallest and largest values, the 10% and 90% quartiles, and the median, mean, and outliers.

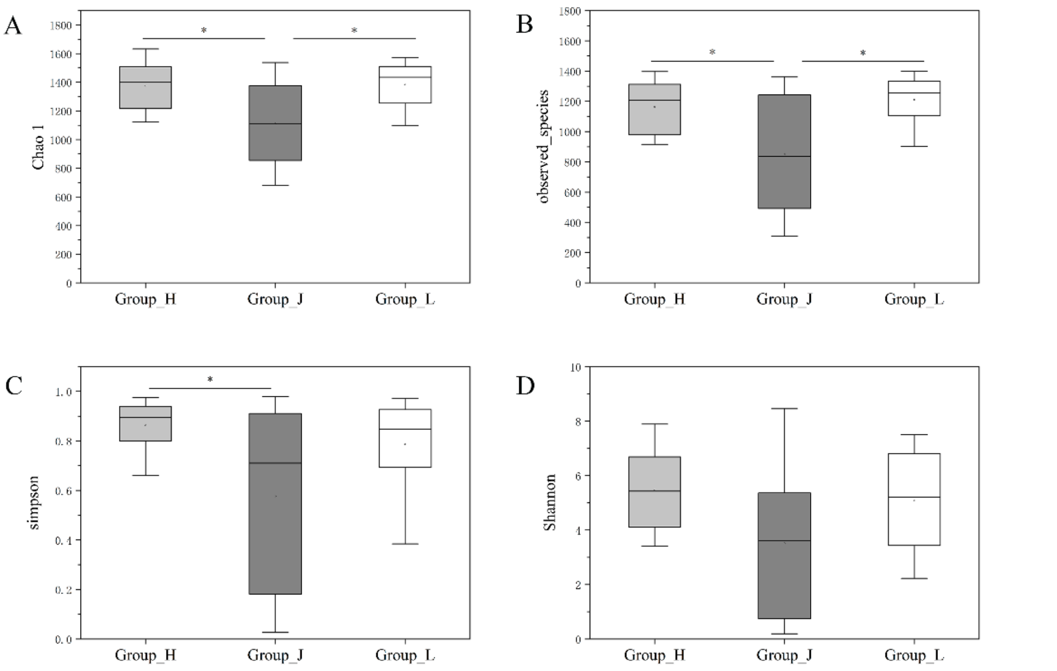

Figure S3. α diversity index of spicy Chinese cabbage samples with different province (Group_J: Jilin Province, Group_L: Liaoning Province, and Group_H: Heilongjiang Province.). (**A**) Chao 1; (**B**) observed_species; (**C**) simpson; (**D**) Shannon. * denote significant differences (P<0.05, ANOVA, Duncan-LSD.). The box plots show the smallest and larg-est values, the 10% and 90% quartiles, and the median, mean, and outliers.

Table S1. The gradient elution program.

elution time/min	mobile phaseA/%	mobile phaseB/%
0	45	55
10	45	55
15	35	65
20	20	80
25	20	80
30	10	90
33	10	90
35	45	55
